# Interaction of Influenza A Nucleoprotein with Host hnRNP-C Is Implicated in Viral Replication

**DOI:** 10.3390/ijms232113613

**Published:** 2022-11-06

**Authors:** Yun-Sang Tang, Wai-Kin So, Ka-Leung Andy Ng, Ka-Pun Chris Mok, Pang-Chui Shaw

**Affiliations:** 1 Centre for Protein Sciences and Crystallography, School of Life Sciences, Faculty of Science, The Chinese University of Hong Kong, Hong Kong SAR, China; 2 Office of University General Education, The Chinese University of Hong Kong, Hong Kong SAR, China; 3 The Jockey Club School of Public Health and Primary Care, Faculty of Medicine, The Chinese University of Hong Kong, Hong Kong SAR, China; 4 Li Ka Shing Institute of Health Sciences, Faculty of Medicine, The Chinese University of Hong Kong, Hong Kong SAR, China; 5 Li Dak Sum Yip Yio Chin R&D Centre for Chinese Medicine, The Chinese University of Hong Kong, Hong Kong SAR, China

**Keywords:** influenza, nucleoprotein, NP, heterogeneous nuclear ribonucleoprotein C, hnRNP-C

## Abstract

The host interactome of influenza viral proteins is ever-expanding. In this work, we report the identification of host heterogeneous nuclear ribonucleoprotein C (hnRNP-C) as an interacting partner of influenza A virus nucleoprotein (NP). We confirmed that this interaction exists across different influenza A subtypes and strains. Using biochemical methods, we determined that hnRNP-C interacts with NP via its C-terminal auxiliary domain. Further, we determined that the hnRNP-C is a negative regulator of influenza viral growth. Its interaction with NP is implicated in the promotion of host cell apoptosis during viral infection. It is the first time that the interaction between influenza nucleoprotein and host heterogeneous nuclear ribonucleoprotein C is characterized in detail. Overall, these findings not only characterize the interaction between NP and its host interacting partner hnRNP-C but also clarify the functional significance of this interaction. This work may lead to a new therapeutic target for the development of anti-influenza drugs.

## 1. Introduction

The influenza virus, an infamous respiratory virus pathogenic to humans, belongs to the orthomyxoviridae family, and its genome contains eight negative, single-stranded RNA gene segments wrapped along the ribonucleoproteins, encoding at least thirteen proteins and polypeptides including the nucleoprotein (NP), which performs multiple structural and functional roles, such as the formation of viral ribonucleoprotein (RNP) with PA, PB1 and PB2, and the encapsidation of viral RNA. NP is an important protein for the stabilization of positive-sense complementary RNA during the replication process, via the formation of a complementary ribonucleoprotein (cRNP) [1]. It also interacts with other viral factors such as the M1 protein, so it forms a “daisy chain” with M1 and NS2 to facilitate viral RNP nuclear export [2].

NP has also been found to interact with multiple host factors including a-actinin 4, TRIM22, clusterin, heat shock protein 40, and nuclear factor 90 [3,4,5,6,7]. The interaction of NP with host importin alpha mediates the nuclear import of viral RNP and progeny NP molecules during the transcription process and is facilitated by the binding of importin alpha to two major nuclear localization signals, respectively, on NP [8,9]. NP homo-oligomerizes by inserting its flexible tail loop into a groove on the body domain, forming an E339–R416 salt bridge [10,11]. Additionally, we previously identified that the positive residues R65 and K87 and two positive grooves are essential for NP binding to viral RNA, while residue R152 is critical for NP–PB2 interaction [11,12]. Hence, NP utilizes different structural motifs to support its versatile biological roles.

hnRNP-C is a member of a family of more than 20 heterogeneous nuclear ribonucleoproteins (hnRNPs). It resides in the host cell nucleus and is involved in RNA processing such as mRNA splicing, mRNA retention and packaging, maintenance of mRNA stability, and mRNA export. It has also been reported to be involved in translation initiation by binding to internal ribosomal entry sites on mRNA [13,14]. hnRNP-C exists in the host as two spliceoforms C1 and C2, where the C2 spliceoform has 13 additional amino acids at the RNA-binding domain but with no functional differences [15]. Host hnRNP-C exists as a (C1)_3_(C2) heterotetramer.

hnRNP-C is implicated in numerous viral infections, including poliovirus, hepatitis delta virus, Ebola virus, dengue virus, the human papillomavirus type 16, MERS-CoV, and SARS-CoV-2 [16,17,18,19,20]. Recently, influenza NP was shown to interact with hnRNP-A2/B1, hnRNP-AB, and hnRNP-A1, which are also members of the family of more than 20 hnRNPs [21,22,23], while hnRNP-C was suggested to interact with influenza NS1 [24]. NP was found to interact with the glycine-rich domain of hnRNP-A2/B1, which was shown to be a positive regulator of RNP activity [21]. Wang et al. showed that NP binds to hnRNP-AB to block its Gly motif, thereby obscuring the interaction between mRNA nuclear export factors and eventually leading to the nuclear retention of viral mRNA [22]. As such, viral replication is repressed. Kaur et al. then demonstrated that hnRNP-A1 interacts with NP so that it negatively regulates the NP expression level and viral growth via an unknown pathway [23]. Hence, the binding of different hnRNPs to NP may lead to either up- or downregulation of RNP activity. In this report, we show that hnRNP-C negatively regulates RNP activity and viral growth by interacting with NP.

## 2. Results

### 2.1. Host Heterogeneous Nuclear Ribonucleoprotein C (hnRNP-C) Was Identified as an Interacting Partner of NP

In order to identify the interacting partners of NP, we immobilized purified H5N1 NP protein onto N-hydroxysuccinimide (NHS) beads and allowed H292 lysates to flow through the beads. The proteins captured by NP were resolved via 2D electrophoresis. hnRNP-C was found, along with nucleophosmin (NPM) and beta-actin, which were previously identified as the interacting partners of NP [25,26].

### 2.2. NP and hnRNP-C Directly Bind to One Another 

**Figure 1:**
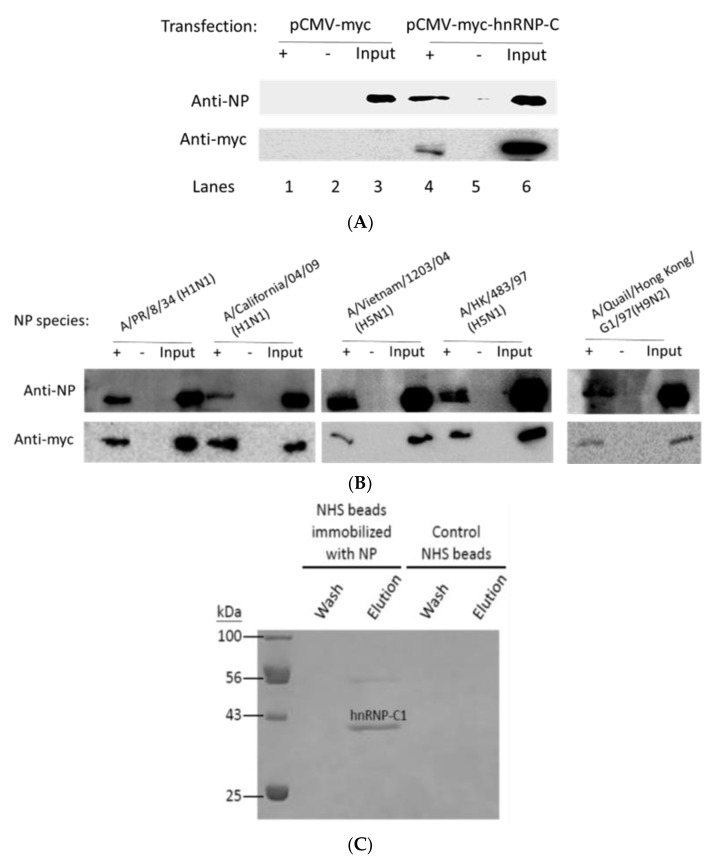
(**A**) HEK293T cells were transfected with myc-hnRNP-C and infected with A/WSN/1933 (H1N1) virus at MOI 0.05 for 24 h. NP from infection was co-immunoprecipitated by myc-tagged hnRNP-C (lane 4) but not by empty vector control (lane 1) or antibody controls (lanes 2 and 5). Inputs were loaded (0.2× volume) as lanes 3 and 6 in the figure; (**B**) HEK293T cells were co-transfected with myc-hnRNP-C and various untagged NP species in pcDNA3. hnRNP-C was immunoprecipitated by anti-myc and NP was detected using NP anti-serum. NP species used in this experiment include A/PR/8/34(H1N1), A/California/04/09(H1N1), A/Vietnam/1203/04(H5N1), A/Hong Kong/483/97(H5N1), and A/Quail/Hong Kong/G1/97(H9N2). (**A**,**B**) +: myc antibody added; −: negative controls without myc antibody; (**C**) purified H5 NP was immobilized on NHS-Sepharose, and purified hnRNP-C1 was allowed to pass through the beads. After washing, hnRNP-C1 could be eluted by 2 M NaCl, indicating in vitro binding between the two proteins. No hnRNP-C1 could be eluted from control NHS beads (activated–inactivated beads without NP), ruling out the possibility of non-specific binding.

To confirm the NP–hnRNP-C interaction in a cellular context upon infection, HEK293T cells overexpressing myc-tagged hnRNP-C1 were infected by the A/WSN/1933(H1N1) virus. Viral NP was co-immunoprecipitated by myc-tagged hnRNP-C1 (Figure 1A, lane 4) but not the empty vector control (Figure 1A, lane 1) or the antibody control (Figure 1A, lanes 2, 5). We then tested the interaction with hnRNP-C with a co-immunoprecipitation experiment using NP from various H1, H5, and H9 influenza strains on the pcDNA3 backbone. The five additional strains tested included A/PR/8/34(H1N1), A/California/04/09(H1N1), A/Vietnam/1203/04(H5N1), A/Hong Kong/483/97(H5N1), and A/Quail/Hong Kong/G1/97(H9N2). Since all of the five NP were co-immunoprecipitated by hnRNP-C, as shown in Figure 1B, this interaction appears to be ubiquitous among influenza A subtypes. We also performed an in vitro NHS pull-down assay using NP (from A/HK/483/97 (H5N1)) and hnRNP-C1 proteins. After extensive washing, hnRNP-C1 was eluted from immobilized NP, demonstrating the interaction in vitro (Figure 1C). This binding was not due to non-specific bead interaction because no hnRNP-C1 was eluted from activated–inactivated control beads.

### 2.3. Characterization of NP–hnRNP-C Interaction

**Figure 2:**
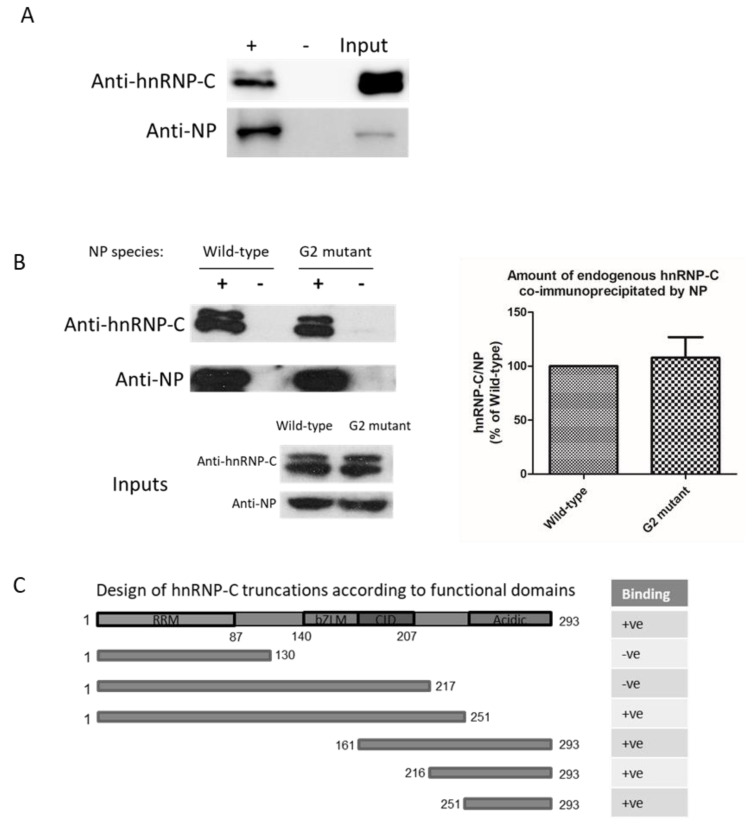
(**A**) myc-tagged H5 NP was used to co-immunoprecipitate endogenous hnRNP-C in the presence of RNase A. NP–hnRNP-C interaction was not abolished with RNase A treatment. +: myc antibody added; −: negative controls without myc antibody; (**B**) wild-type and H5 NP G2 mutant co-immunoprecipitated comparable amounts of endogenous hnRNP-C; (**C**) summary of pull-down findings in this work. +ve indicates binding observed for a construct; -ve indicates binding not observed for a construct. Numbers indicate the residue positions of each construct (not on scale). Original gel photos and a schematic figure of hnRNP-C functional domains can be found in Supplementary Figure S1A–D. Abbreviations: RRM, RNA recognition motif; bZLM, basic leucine zipper-like motif; CID, C1–C1 interaction domain; Acidic, acidic auxiliary domain.

Since both NP and hnRNP-C were found to bind RNA [11,27], we proceeded to determine whether RNA is involved in this interaction. Co-immunoprecipitation was performed in the presence of RNase A (to a final concentration of 1 ug/mL) to remove RNA in crude cell lysate. RNA removal did not abolish the interaction between endogenous hnRNP-C1/C2 and myc-tagged H5 NP (Figure 2A). In addition, we previously showed a “G2 mutant” of H5 NP retained only one-fifth of the RNA binding affinity compared with the wild-type [8]. Despite a lower RNA-binding affinity, G2 mutant co-immunoprecipitated a comparable amount of hnRNP-C1/C2 as wild-type protein (Figure 2B). Taking these lines of evidence together, we conclude that RNA is not a necessary condition for the NP–hnRNP-C interaction.

We then set forth to define the NP-binding region on hnRNP-C by constructing various hnRNP-C truncation fragments to evaluate the extent of their in vitro pull-down by NP. Full-length hnRNP-C and its fragments were passed through NP, which was immobilized on NHS-Sepharose beads. For C-terminal truncations, only fragment 1–251 was weakly captured by NP but not the fragments 1–130 and 1–217. For N-terminal truncations, fragments 161–293, 216–293, and 251–293 were all captured by NP to various extents (Figure 2C and Supplementary Figure S1A–C; all numberings refer to the hnRNP-C1 isoform). It could then be summarized that hnRNP-C relies on its C-terminal acidic auxiliary domain for binding to NP. Although the exact functions of this auxiliary domain remain elusive, it was not found to bind RNA due to its highly acidic nature, further suggesting that the NP–hnRNP-C interaction was not RNA-dependent.

### 2.4. hnRNP-C Is Implicated in Influenza Virus Replication

**Figure 3:**
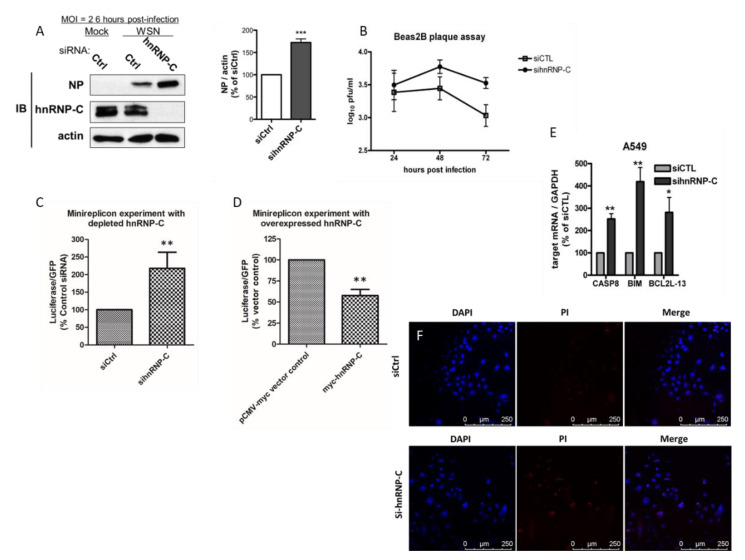
(**A**,**B**) Growth of A/WSN/1933 (H1N1) in hnRNP-C-silenced Beas2B cells was found to be enhanced compared with control Beas2B cells. By Western blotting, we observed a rise in protein level for NP at 6 h post-infection by A/WSN/1933 (H1N1) if endogenous hnRNP-C was transiently knocked down prior to infection. Growth enhancement was exacerbated at later time points, as revealed by plaque assay; (**C**) upon hnRNP-C depletion by hnRNP-C-targeting siRNA, luciferase signal rose to 218% of control siRNA, reflecting an enhanced polymerase activity; (**D**) when hnRNP-C was overexpressed, a drop in luciferase signal was observed, reflecting a suppression in polymerase activity, further suggesting hnRNP-C to be a negative regulator of IAV; (**E**) through real-time PCR, expression of pro-apoptotic factors caspase 8 (CASP8), BCL2-like 11 apoptosis facilitator (BCL2L11), and BCL2-like 13 apoptosis facilitator (BCL2L13) were all upregulated upon transient knockdown of endogenous hnRNP-C; (**F**) A549 transfected with either control or specific hnRNP-C siRNA were double-stained with 4′,6-diamidino-2-phenylindole (DAPI) and propidium iodide (PI). More cells were PI-stained upon hnRNP-C depletion. Magnification at 20×. For panels A–E, * *p* < 0.05, ** *p* < 0.01, *** *p* < 0.001 in two-tailed Student’s *t*-test, *n* = 3.

In order to gain insights into how host hnRNP-C affects the viral cycle of the influenza virus, the growth kinetics of the virus was investigated in Bease2B cells with endogenous hnRNP-C1/-C2 depleted by specific siRNA and then infected with A/WSN/1933(H1N1). It could be shown that when hnRNP-C was transiently knocked down, viral growth was enhanced at as early a time point as 6 h post-infection (Figure 3A, left panels), as reflected by an increased amount of NP being detected. In fact, our data with A549 (Supplementary Figure S2a) showed similar enhancement in viral growth upon hnRNP-C depletion at 6 h post-infection. The enhancement in viral growth was exacerbated at later time points such as at 48 h and 72 h post-infection, when viral titers in cells with depleted hnRNP-C were significantly higher than those in cells transfected with control siRNA (Figure 3B), further suggesting that hnRNP-C is a negative regulator of influenza viral growth. When hnRNP-C was partially depleted in HEK293T, the expression of luciferase reporter was enhanced to 218%, showing an enhancement of polymerase activity (Figure 3C and Supplementary Figure S2b). In contrast, when hnRNP-C was overexpressed, polymerase activity was suppressed, as reflected by a lower luciferase signal (58% relative to empty vector control) (Figure 3D). These results suggest that the modulation of RNP activity by hnRNP-C should contribute to affecting viral growth. In order to shed more light on the functional implications of the NP–hnRNP-C interaction, we transiently silenced hnRNP-C in A549 cells. By doing so, pro-apoptotic factors caspase 8 (CASP8), BCL2-like 11 apoptosis facilitator (BCL2L11), and BCL2-like 13 apoptosis facilitator (BCL2L13) were upregulated (Figure 3E). Hence, hnRNP-C itself is anti-apoptotic. To further confirm apoptosis, we stained A549 cells transfected with control and specific hnRNP-C siRNA with both 4′,6-diamidino-2-phenylindole (DAPI) and propidium iodide (PI). In the cells depleted with hnRNP-C, more cells could be stained with propidium iodide, suggesting an elevated level of the apoptotic event. These results prompted us to postulate that the enhancement of RNP activity and viral growth upon hnRNP-C depletion may be related to host cell apoptosis.

### 2.5. Perturbation of NP–hnRNP-C Interaction Reduces Ribonucleoprotein (RNP) Activity

**Figure 4:**
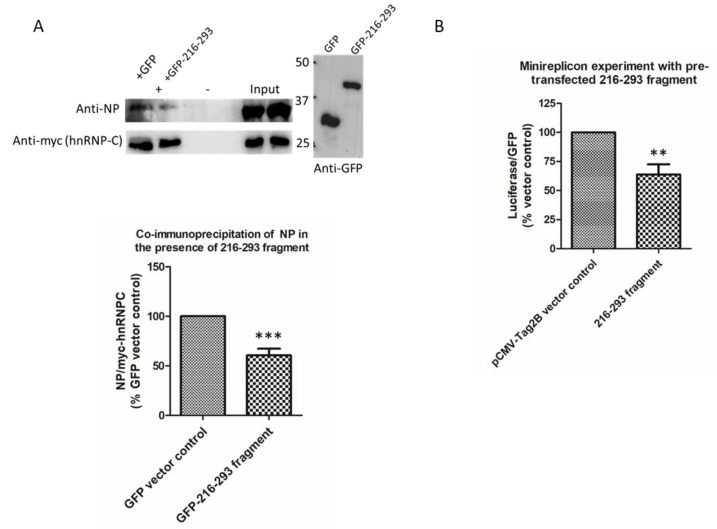
(**A**) Co-immunoprecipitation in HEK293T cells overexpressing GFP (control) or GFP–216–293 fragment. In the presence of GFP–216–293, only 60.5% of it, compared with vector control, could be co-immunoprecipitated by myc–hnRNP-C. ***: *p* < 0.001, two-tailed Student’s *t*-test, error bar represents standard deviation over *n* = 3 experiments; +: myc antibody added; −: negative controls without myc antibody (**B**) Flag 216–293 was co-transfected to HEK293T cells along with the influenza minireplicon. A drop in luciferase signal to 63.8% (**: *p* < 0.01, two-tailed Student’s *t*-test) was observed when compared to pCMV-Tag2B vector control. The error bar represents the standard deviation over *n* = 4 experiments.

To investigate the functional significance of the NP–hnRNP-C interaction, a fragment corresponding to residues 216–293 of hnRNP-C (216–293 fragment) was constructed to perturb the NP–hnRNP-C interaction by competing with the full-length hnRNP-C protein for binding to NP. In the HEK293T cells expressing the 216–293 fragment and infected with the A/WSN/1933(H1N1) virus, full-length myc-tagged hnRNP-C precipitated around 40% less NP than the amount in cells expressing GFP control (Figure 4A), confirming that this fragment is sufficient to interrupt the NP–hnRNP-C interaction. In the influenza minireplicon assay, the 216–293 fragment was able to suppress the reporter luciferase activity to 63.8%, compared with an empty vector negative control, showing that the ribonucleoprotein (RNP) activity had been interrupted. This suggests that the NP–hnRNP-C interaction is important for maintaining RNP activity.

## 3. Discussion

We identified and characterized the interaction between influenza nucleoprotein (NP) and host heterogeneous nuclear ribonucleoprotein C (hnRNP-C) in this work. We found that the NP–hnRNP-C interaction did not involve RNA and that the C-terminal region on the auxiliary domain of hnRNP-C was responsible for NP binding. A C-terminal fragment from hnRNP-C was able to perturb the NP–hnRNP-C complex formation and suppress the influenza RNP activity.

NP has been shown to interact with hnRNP-A2/B1 via its glycine-rich domain but not the RNA-binding domain [21]. Likewise, we discovered in this work that the NP–hnRNP-C interaction was not RNA-dependent and relied on the auxiliary acidic domain. There are 19 aspartic acid and 16 glutamic acid residues spanning the last 78 amino acids of hnRNP-C, rendering up to 45% of the residues in this region negatively charged. Since the surface charge of NP is largely positive [11,12], it is possible that multiple contact sites actually exist within both proteins. We showed that the auxiliary acidic domain of hnRNP-C is sufficient to suppress RNP activity, and we attributed this to the perturbation of the NP–hnRNP-C interaction. There exists, however, the possibility that the binding of the acidic domain to NP masks its surface positive charges so that it is no longer available for binding to other viral or host factors.

NP is the most abundant protein component of the ribonucleoprotein (RNP), which is responsible for the transcription and replication of viral proteins. Hence, RNP activity would directly correlate with viral fitness. Using a minigenome luciferase reporter assay, we were able to investigate RNP activity as luciferase signal readout. In this assay, PA, PB1, PB2, and NP subunits were co-transfected into HEK293T cells along with the luciferase reporter, driven by an influenza promoter. An EGFP plasmid driven by a CMV promoter was also transfected as an internal control. A minigenome RNP was thereby reconstituted in cells for the transcription of the luciferase gene. Normalized luciferase signal served as a reflection of RNP activity. Utilizing this system, we demonstrated suppressed RNP activity upon hnRNP-C overexpression and, in contrast, enhanced activity during hnRNP-C depletion.

hnRNP-C was reported to be involved in various viral infection conditions. During poliovirus infection, it interacts with the 3′-end of the negative RNA strand and promotes positive-strand synthesis [28]. hnRNP-C was also shown to interact with the small delta antigen of the hepatitis delta virus so that its depletion leads to decreased expression of this viral protein [18]. On the other hand, the Ebola virus VP24 competes with hnRNP-C for karyopherin alpha 1, leading to the relocalization of hnRNP-C from the nucleus to the cytoplasm [19]. Recently, it was found that hnRNP-C binds the 3′-untranslated region on human papillomavirus Type 16 mRNA to activate downstream splicing at the 5′ splice site [29]. More recently, hnRNP-C was shown to perturb host circular RNA–cognate mRNA pairs during the infection of SARS-CoV, MERS-CoV, and SARS-CoV-2 [20]. All in all, hnRNP-C is a versatile protein that is involved in different stages of viral infection.

In our work, we showed that hnRNP-C itself is a negative regulator of RNP activity and viral growth. However, the NP–hnRNP-C interaction is indispensable for proper RNP activity. These two results may seem contradictory at first glance. Nevertheless, based on our findings, we postulate that the influenza NP exploited hnRNP-C to modulate host apoptosis. Consistent with this postulation, it has been reported that the apoptosis-induced enlargement of nuclear pores would facilitate progeny RNP nuclear export [30]; therefore, apoptosis is important for proper RNP function. NP was reported to be a pro-apoptotic viral protein. Knocking down NP reduced apoptosis induced by the H1N1pdm2009 stain [31]. On the other hand, hnRNP-C was found to promote the expression of the X-linked inhibitor of apoptosis protein (XIAP), a well-studied inhibitor of caspase 3, 7, and 9 [32], by binding to the internal ribosomal entry site (IRES) of XIAP mRNA and promoting its translation [14], meaning that its presence is anti-apoptotic. In our work, the knockdown of hnRNP-C led to the upregulation of pro-apoptotic factors, as tested by RT-qPCR. In fact, this is also consistent with our microarray data, which show the upregulation of pro-apoptotic factors and downregulation of anti-apoptotic factors after hnRNP-C depletion (Supplementary Table S2). The co-transfection of a C-terminal fragment of hnRNP-C that was able to interrupt the NP–hnRNP-C binding led to the suppression of RNP activity. This implies that the NP–hnRNP-C interaction is important for the RNP to function properly. We also showed that hnRNP-C is a negative regulator of viral growth (i.e., the knockdown of hnRNP-C enhanced viral growth). Taken altogether, we propose that upon infection, NP occupies hnRNP-C and renders it less available for XIAP translation, thereby indirectly promoting host apoptosis and thus RNP export.

In summary, we characterized, for the first time, the NP–hnRNP-C interaction, and these results provide a better understanding of NP host interactome and give additional insights as to how the influenza A virus acts upon the host cell apoptosis machinery.

## 4. Materials and Methods

### 4.1. Plasmids

pRHisMBP-NP [11] and the plasmids used for the luciferase experiment [33] were described previously. pET28a was used for subcloning of hnRNP-C1 or its fragments and was obtained commercially from Novagen, Madison, Wisconsin, USA. pETZ2-1a was obtained under a material transfer agreement with EMBL. pCMV-myc and pEGFP-C1 were obtained commercially from Clontech, Takara Bio, Shiga, Japan. pCMV-Tag2B was purchased from Stratagene, San Diego, CA, USA.

### 4.2. Cells

A549, Beas2B, H292, MDCK, and HEK293T cells were obtained from ATCC. A549 and H292 were maintained in RPMI-1640 medium. MDCK was maintained in a minimal essential medium (MEM). HEK293T was maintained in a Dulbecco’s modified Eagle’s medium (DMEM). Beas2B was maintained in a DMEM with Ham’s F-12 supplement. All media were supplemented with 10% heat-inactivated fetal bovine serum (FBS), 100 IU/mL penicillin, and 100 ug/mL streptomycin. The cells were cultured at 37 °C under 5% CO_2_. All culture media and supplements were obtained from Gibco unless otherwise stated.

### 4.3. Antibodies

Anti-myc (Cell Signalling), anti-actin (Genscript), and anti-GFP (JL-8) (Takara Bio USA) were obtained commercially. Horseradish peroxidase (HRP)-conjugated anti-mouse, and anti-rabbit secondary antibodies were purchased from Bio-rad, Hercules, CA, USA. Anti-hnRNP-C was purchased from Santa Cruz Biotechnology, Dallas, TX, USA. NP anti-serum was raised in-house and described previously [34].

### 4.4. Expression of NP, Pull-Down, and Mass Spectrometric Analysis

The nucleoprotein of A/HK/156/97 (H5N1) was expressed as a maltose binding protein (MBP) fusion protein as described previously [8]. After RNase A and thrombin treatment, purified NP was coupled to NHS beads. The crude lysate of H292 cells was allowed to flow through immobilized NP. After extensive washing, bound proteins were eluted with 2 M NaCl, separated through 2D electrophoresis, and visualized via silver staining. Surplus spots compared with the control (activated–deactivated NHS beads without immobilized NP) were excised for MALDI–TOF MS/MS analysis. A combined (MS + MS/MS) analysis was carried out against the NCBI non-redundant database using the ProteinScape 3.0 software from Bruker, Billerica, MA, USA.

### 4.5. Virus Infection

For infection experiments, the culture medium was aspirated, and the cells were washed twice with PBS. The A/WSN/1933 (H1N1) virus was diluted in a serum-free medium and was used for infection at the multiplicity of infection (MOI) as stated. The serum-free medium was used as a mock infection control. After 1 h incubation at room temperature, the virus was replaced with a fresh serum-free medium, and the cells were incubated at 37 °C for virus propagation. Supernatants were collected at 24 h post-infection for titer determination or further experiment.

### 4.6. Co-Immunoprecipitation

Co-immunoprecipitation was performed in HEK293T cells by transfecting relevant plasmids, as stated in the text. The cells were lysed through sonication in an IP buffer (20 mM Tris, 150 mM NaCl, 1 mM EDTA, 0.5% Triton-X-100). After clearance of cell debris via centrifugation, immunoprecipitation was performed by the addition of appropriate antibodies at room temperature for 4 h or at 4 °C overnight, followed by incubation with Protein A beads for 1 h. After extensively washing with the IP buffer, the beads were boiled in SDS-loading dye and subjected to SDS–PAGE and Western blotting. In the case of infection as a source of NP, myc-tagged hnRNP-C1 was transfected to HEK293T. Then, 12–16 h later, the cells were washed with PBS and changed to a serum-free DMEM, after which they were infected with the A/WSN/1933 (H1N1) virus at an MOI of 0.01 for 12–16 h. To test for the effect of the hnRNP-C fragment of residues 216–293, the plasmid encoding GFP–216–293 fragment or the control pEGFP-C1 was pre-transfected to HEK293T 24 h prior to infection. myc-tagged hnRNP-C was expressed in HEK293T separately. At 24 h post-infection, the cells were harvested, sonicated, and cleared. Lysates with myc-hnRNP-C were mixed with the lysates with GFP or GFP–216–293 to allow for immunoprecipitation at 4 °C overnight.

### 4.7. In Vitro NHS Pull-Down 

hnRNP-C and its truncations were expressed in the pET28a vector or in the pETZ2_1a vector (for fragment 251-293 only) in BL21(DE3)pLysS cells. A list of primers used for cloning these truncations is included in Supplementary Table S1. Protein expression was induced by IPTG with 0.4 mM at OD600 = 0.8–1.0 at 25 °C with agitation at 210 rpm. The cells were collected after 6 h through centrifugation and then collected on ice in a lysis buffer (20 mM Tris pH7, 1M NaCl, 20 mM imidazole, 2 mM beta-mercaptoethanol, and 1 mM PMSF). The whole-cell lysate was then centrifuged at 20,000 rpm for 1 h at 4 °C) before being loaded onto a nickel affinity column. The column was extensively washed with 20 mM Tris pH7, 500 mM, and 50 mM imidazole. Elution was achieved by 20 mM Tris pH7, 200 mM NaCl supplemented with 500 mM imidazole. RNase A was added to remove residual RNA from cells at a final concentration of 1 U/mL at 4 °C overnight. The proteins were then buffer-exchanged to 20 mM Tris pH8, 300 mM NaCl, and 10% glycerol. For the in vitro pull-down assay, purified NP (in a phosphate buffer) was immobilized onto N-hydroxysuccinimide (NHS)-coupled beads (GE Healthcare, now Cytiva) according to the manufacturer’s protocol. A buffer was added to treat a portion of the beads as the negative control. NP-coupled beads were incubated with hnRNP-C or truncations at room temperature for 2 h with agitation. The beads were washed extensively with 20 mM Tris pH8, and 300 mM NaCl and eluted with two fractions of 2 M NaCl. The eluted proteins were resolved in SDS–PAGE and were subsequently stained with Coomassie Blue.

### 4.8. Reverse Transcription qPCR (RT-qPCR)

The culture medium was removed, and the cells were collected in 500 μL TRIzol Reagent (Invitrogen). The total RNA extracted was used in first-strand DNA (cDNA) synthesis using M-MLV (Genesys, Camberley, UK) for real-time PCR, according to the manufacturer’s protocol. Real-time PCR was performed in Applied Biosystems 7300 (Perkin Elmer) using the following primers: hnRNP-C, forward 5′-GCA GGT GTG AAA CGA TCT GC-3′ and reverse 5′-TTA CTT TCC AGA CTT GGA AGA TCC-3′; CASP8, forward 5′-AAG CAA ACC TCG GGG ATA CT-3′ and reverse 5′-GGG GCT TGA TCT CAA AAT GA-3′; BIM, forward 5′-GAG ATA TGG ATC GCC CAA GA-3′ and reverse 5′-CAA TGC ATT CTC CAC ACC AG-3′; BCL2L13, forward 5′-CTG AGC CAG CCA GTG ACA TA-3′ and reverse 5′-TCC AGG GTA TTC CTC CTC CT-3′; GAPDH, forward 5′-GAG TCA ACG GAT TTG GTC GT-3′ and reverse 5′-GAC AAG CTT CCC GTT CTC AG-3′. PCR reactions constituted of a 3 min hot start at 95 °C, followed by 40 cycles of denaturation at 95 °C for 15 sec and amplification at 60 °C for 30 s, and extension at 72 °C for 15 s. All RT-qPCR results represent the mean from at least three independent experiments. The relative quantification of mRNA levels was performed with the comparative Ct method using GAPDH as the reference gene and the 2^−∆∆Ct^ method.

### 4.9. Knockdown of hnRNP-C

For the endogenous hnRNP-C knockdown, Beas-2B or HEK293T cells were transfected with human hnRNP-C1/C2 siRNA (Santa Cruz Biotechnology) using Lipofectamine RNAiMAX (Invitrogen, Burlington, ON, USA). Non-targeting siRNA (Santa Cruz Biotechnology, Dallas, TX, USA) was used as a transfection control. For the transfection of one well on a 24-well plate, 0.06 nmol of siRNA was transfected.

### 4.10. Minigenome Luciferase Reporter Assay

The system has been described previously [20], which was used with modifications in this work. Briefly, 0.5 µg of each of pcDNA3-PA, pcDNA3-PB1, pcDNA3-PB2, pEGFP-C1, and pPol-luci-RT and 1 µg of pcDNA3-NP were transfected to HEK293T cells on a 24-well plate. Where necessary, the cells were co-transfected with the following plasmids alongside RNP components: 0.5 µg pCMV-myc or pCMV-myc-hnRNP-C, for the hnRNP-C perturbation experiment; 2 µg pCMV-Tag2B or pCMV-Tag2B-216–293, which encodes a Flag-tagged fragment of hnRNP-C spanning residues 216–293, for the peptide perturbation experiment. Forty-eight hours post-transfection, the cells were lysed in 50 mM Tris pH 7.8, 150 mM NaCl, 10% glycerol, 1% Triton X-100, 2 mM DTT, and 1 mM EDTA with gentle agitation. In the case of the hnRNP-C knockdown experiment, siRNA was first transfected according to the description above. Then, 24 h later, the transfection of RNP components was performed in fresh DMEM/10%FBS, and the cells were incubated for a further 24 h. The chemiluminescence signal was measured on a ClarioStar spectrophotometer, with a firefly luciferase substrate (Promega, Madison, WI, USA). The chemiluminescence signals were normalized against the GFP signal. The experiment was repeated for four biological replicates. Statistical comparison was performed using Student’s *t*-test in GraphPad Prism.

### 4.11. Fluorescence Staining

A549 cells were pre-seeded on 12 mm diameter glass slides in a 24-well plate. hnRNP-C was depleted by specific siRNA, as described above. The cells were harvested by removal of the medium and washed three times with cold PBS. Diamidino-2-phenylindole (DAPI) at 1 µg/mL was added to each well for 5 min in the dark. Afterward, the cells were washed three times with cold PBS, and propidium iodide at 1 µg/mL was added for staining in the dark for 20 min. The cells were then washed three times with cold PBS and mounted onto microscopy slides using a DAKO mounting medium. The cells were visualized with a Leica TCS SP8 Confocal Microscope System. For comparison purposes, acquisition parameters remained identical for all slides.

### 4.12. Western Blot

The samples were resolved on SDS–PAGE gels and electrotransferred to a PVDF membrane. After blocking for 1 h with 5% non-fat dry milk in a TBS-T buffer (20 mM Tris-HCl, pH 7.4, 150 mM NaCl, 0.1% Tween-20), the blots were probed overnight at 4 °C with primary antibodies as stated. The blots were then incubated with a horseradish peroxidase-conjugated secondary antibody for 1 h followed by detection with ECL chemiluminescence reagent (Amersham, Arlington Heights, IL, USA) and exposure on X-ray films or detection with a ChemiDocMP+ imager (Bio-rad).

## Data Availability

Not applicable.

## Supplementary Materials

The following supporting information can be downloaded at: https://www.mdpi.com/article/10.3390/ijms232113613/s1, Figure S1: Pull-down results of hnRNP-C truncations using immobilized NP; Figure S2a: Enhancement of viral growth in A549 cells at 6 h post-infection, as revealed by increased NP amount in Western Blots; Figure S2b: Representative Western blot image for partial depletion of hnRNP-C in HEK293T cells by specific siRNA. Table S1: Primers for cloning. Table S2: Summary of microarray analysis.

## References

[1] Vreede F.T., Ng A.K.-L., Shaw P.C., Fodor E. (2011). Stabilization of Influenza Virus Replication Intermediates Is Dependent on the RNA-Binding but Not the Homo-Oligomerization Activity of the Viral Nucleoprotein. J. Virol..

[2] Bui M., Wills E.G., Helenius A., Whittaker G.R. (2000). Role of the Influenza Virus M1 Protein in Nuclear Export of Viral Ribonucleoproteins. J. Virol..

[3] Sharma K., Tripathi S., Ranjan P., Kumar P., Garten R., Deyde V., Katz J.M., Cox N.J., Lal R.B., Sambhara S. (2011). Influenza A Virus Nucleoprotein Exploits Hsp40 to Inhibit PKR Activation. PLoS ONE.

[4] Sharma S., Mayank A.K., Nailwal H., Tripathi S., Patel J.R., Bowzard J.B., Gaur P., Donis R.O., Katz J.M., Cox N.J. (2014). Influenza A Viral Nucleoprotein Interacts with Cytoskeleton Scaffolding Protein α-Actinin-4 for Viral Replication. FEBS J..

[5] Di Pietro A., Kajaste-Rudnitski A., Oteiza A., Nicora L., Towers G.J., Mechti N., Vicenzi E. (2013). TRIM22 Inhibits Influenza A Virus Infection by Targeting the Viral Nucleoprotein for Degradation. J. Virol..

[6] Tripathi S., Batra J., Cao W., Sharma K., Patel J.R., Ranjan P., Kumar A., Katz J.M., Cox N.J., Lal R.B. (2013). Influenza A Virus Nucleoprotein Induces Apoptosis in Human Airway Epithelial Cells: Implications of a Novel Interaction between Nucleoprotein and Host Protein Clusterin. Cell Death Dis..

[7] Wang P., Song W., Mok B.W.Y., Zhao P., Qin K., Lai A., Smith G.J.D., Zhang J., Lin T., Guan Y. (2009). Nuclear Factor 90 Negatively Regulates Influenza Virus Replication by Interacting with Viral Nucleoprotein. J. Virol..

[8] Wu W., Sankhala R.S., Florio T.J., Zhou L., Nguyen N.L.T., Lokareddy R.K., Cingolani G., Panté N. (2017). Synergy of Two Low-Affinity NLSs Determines the High Avidity of Influenza A Virus Nucleoprotein NP for Human Importin α Isoforms. Sci. Rep..

[9] Nakada R., Hirano H., Matsuura Y. (2015). Structure of Importin-α Bound to a Non-Classical Nuclear Localization Signal of the Influenza A Virus Nucleoprotein. Sci. Rep..

[10] Ye Q., Krug R.M., Tao Y.J. (2006). The Mechanism by Which Influenza A Virus Nucleoprotein Forms Oligomers and Binds RNA. Nature.

[11] Ng A.K.L., Zhang H., Tan K., Li Z., Liu J.H., Chan P.K.S., Li S.M., Chan W.Y., Au S.W.N., Joachimiak A. (2008). Structure of the Influenza Virus A H5N1 Nucleoprotein: Implications for RNA Binding, Oligomerization, and Vaccine Design. FASEB J..

[12] Tang Y.S., Xu S., Chen Y.W., Wang J.H., Shaw P.C. (2021). Crystal Structures of Influenza Nucleoprotein Complexed with Nucleic Acid Provide Insights into the Mechanism of RNA Interaction. Nucleic Acids Res..

[13] Lewis S.M., Veyrier A., Hosszu Ungureanu N., Bonnal S., Vagner S., Holcik M. (2007). Subcellular Relocalization of a Trans-Acting Factor Regulates XIAP IRES-Dependent Translation. Mol. Biol. Cell.

[14] Holcík M., Gordon B.W., Korneluk R.G. (2003). The Internal Ribosome Entry Site-Mediated Translation of Antiapoptotic Protein XIAP Is Modulated by the Heterogeneous Nuclear Ribonucleoproteins C1 and C2. Mol. Cell. Biol..

[15] Burd C.G., Swanson M.S., Görlach M., Dreyfuss G. (1989). Primary Structures of the Heterogeneous Nuclear Ribonucleoprotein A2, B1, and C2 Proteins: A Diversity of RNA Binding Proteins Is Generated by Small Peptide Inserts. Proc. Natl. Acad. Sci. USA.

[16] Dechtawewat T., Songprakhon P., Limjindaporn T., Puttikhunt C., Kasinrerk W., Saitornuang S., Yenchitsomanus P., Noisakran S. (2015). Role of Human Heterogeneous Nuclear Ribonucleoprotein C1/C2 in Dengue Virus Replication. Virol. J..

[17] Gustin K.E., Sarnow P. (2001). Effects of Poliovirus Infection on Nucleo-Cytoplasmic Trafficking and Nuclear Pore Complex Composition. EMBO J..

[18] Casaca A., Fardilha M., da Cruz e Silva E., Cunha C. (2011). The Heterogeneous Ribonuclear Protein C Interacts with the Hepatitis Delta Virus Small Antigen. Virol. J..

[19] Shabman R.S., Gulcicek E.E., Stone K.L., Basler C.F. (2011). The Ebola Virus VP24 Protein Prevents HnRNP C1/C2 Binding to Karyopherin A1 and Partially Alters Its Nuclear Import. J. Infect. Dis..

[20] Zhang X., Chu H., Chik K.K.H., Wen L., Shuai H., Yang D., Wang Y., Hou Y., Yuen T.T.T., Cai J.P. (2022). HnRNP C Modulates MERS-CoV and SARS-CoV-2 Replication by Governing the Expression of a Subset of CircRNAs and Cognitive MRNAs. Emerg. Microbes Infect..

[21] Chang C.-K., Chen C.-J., Wu C.-C., Chen S.-W., Shih S.-R., Kuo R.L. (2017). Cellular HnRNP A2/B1 Interacts with the NP of Influenza A Virus and Impacts Viral Replication. PLoS ONE.

[22] Wang X., Lin L., Zhong Y., Feng M., Yu T., Yan Y., Zhou J., Liao M. (2021). Cellular HnRNPAB Binding to Viral Nucleoprotein Inhibits Flu Virus Replication by Blocking Nuclear Export of Viral MRNA. iScience.

[23] Kaur R., Batra J., Stuchlik O., Reed M.S., Pohl J., Sambhara S., Lal S.K. (2022). Heterogeneous Ribonucleoprotein A1 (HnRNPA1) Interacts with the Nucleoprotein of the Influenza a Virus and Impedes Virus Replication. Viruses.

[24] Wu X., Wang S., Yu Y., Zhang J., Sun Z., Yan Y., Zhou J. (2013). Subcellular Proteomic Analysis of Human Host Cells Infected with H3N2 Swine Influenza Virus. Proteomics.

[25] Mayer D., Molawi K., Martínez-Sobrido L., Ghanem A., Thomas S., Baginsky S., Grossmann J., García-Sastre A., Schwemmle M. (2007). Identification of Cellular Interaction Partners of the Influenza Virus Ribonucleoprotein Complex and Polymerase Complex Using Proteomic-Based Approaches. J. Proteome Res..

[26] Portela A., Digard P. (2002). The Influenza Virus Nucleoprotein: A Multifunctional RNA-Binding Protein Pivotal to Virus Replication. J. Gen. Virol..

[27] Cieniková Z., Damberger F.F., Hall J., Allain F.H.T., Maris C. (2014). Structural and Mechanistic Insights into Poly(Uridine) Tract Recognition by the HnRNP C RNA Recognition Motif. J. Am. Chem. Soc..

[28] Brunner J.E., Nguyen J.H.C., Roehl H.H., Ho T.V., Swiderek K.M., Semler B.L. (2005). Functional Interaction of Heterogeneous Nuclear Ribonucleoprotein C with Poliovirus RNA Synthesis Initiation Complexes. J. Virol..

[29] Dhanjal S., Kajitani N., Glahder J., Mossberg A.K., Johansson C., Schwartz S. (2015). Heterogeneous Nuclear Ribonucleoprotein C Proteins Interact with the Human Papillomavirus Type 16 (HPV16) Early 3’-Untranslated Region and Alleviate Suppression of HPV16 Late L1 MRNA Splicing. J. Biol. Chem..

[30] Mühlbauer D., Dzieciolowski J., Hardt M., Hocke A., Schierhorn K.L., Mostafa A., Müller C., Wisskirchen C., Herold S., Wolff T. (2015). Influenza Virus-Induced Caspase-Dependent Enlargement of Nuclear Pores Promotes Nuclear Export of Viral Ribonucleoprotein Complexes. J. Virol..

[31] Yu K., Ren Y., Zhang X., Qiao T., Liu Z., Shi J., Wang Y. (2017). ShRNA-Mediated NP Knockdown Inhibits the Apoptosis of Cardiomyocytes Induced by H1N1pdm2009 Influenza Virus. Mol. Med. Rep..

[32] Deveraux Q.L., Leo E., Stennicke H.R., Welsh K., Salvesen G.S., Reed J.C. (1999). Cleavage of Human Inhibitor of Apoptosis Protein XIAP Results in Fragments with Distinct Specificities for Caspases. EMBO J..

[33] Lo C.Y., Li O.T.W., Tang W.P., Hu C., Wang G.X., Ngo J.C.K., Wan D.C.C., Poon L.L.M., Shaw P.C. (2018). Identification of Influenza Polymerase Inhibitors Targeting C-Terminal Domain of PA through Surface Plasmon Resonance Screening. Sci. Rep..

[34] Szeto W.C., Hsia H.-P., Tang Y.S., Shaw P.-C. (2020). Interaction between Influenza A Virus Nucleoprotein and PB2 Cap-Binding Domain Is Mediated by RNA. PLoS ONE.

