# Supplementary material for: Interaction of Influenza A Nucleoprotein with Host hnRNP-C Is Implicated in Viral Replication
Source: Int J Mol Sci. 2022 Nov 6;23(21):13613. doi: 10.3390/ijms232113613 (PMC9655074; doi:10.3390/ijms232113613)
Supplement: Supplementary file 1 [file ijms-23-13613-s001.zip › ijms-1973522-supplementary.pdf]

Figure S1:

Pull-down results of hnRNP-C truncations using immobilized NP

Supplementary Figure S1A

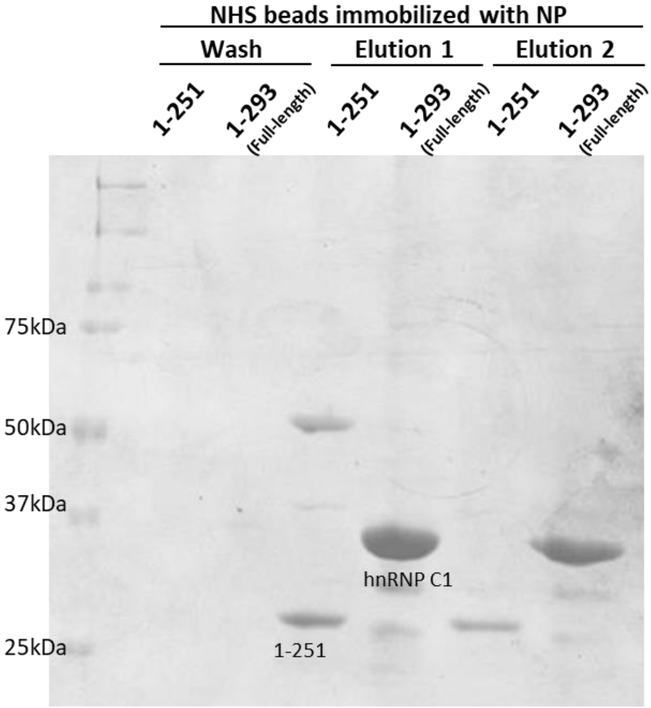

Supplementary Figure S1B

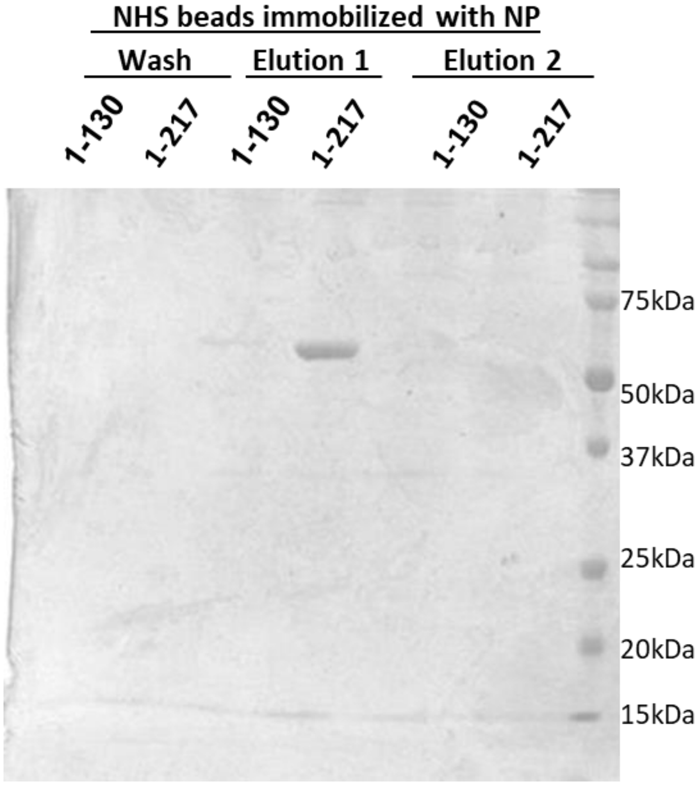

Supplementary Figure S1C

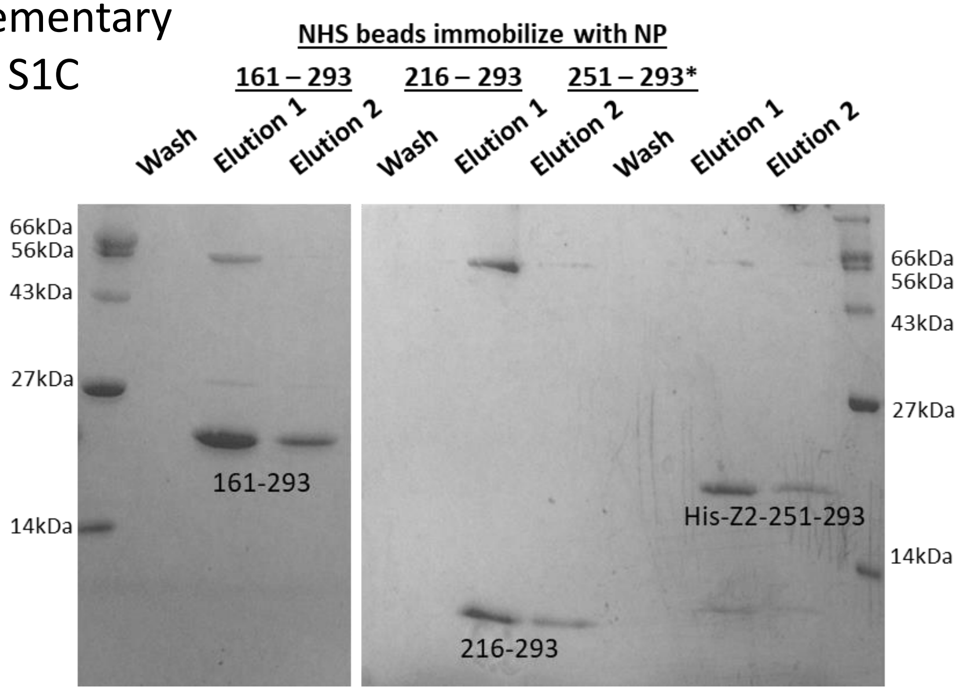

(* 251-293 fragment with His-Z2 tag uncut.)

Supplementary Figure S1D

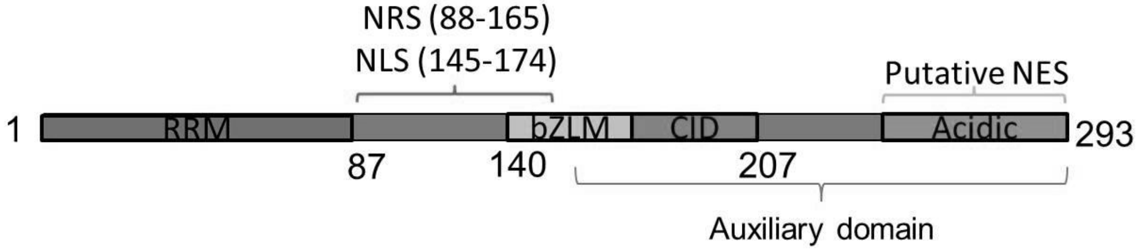

RRM: RNA recognition motif
bZLM: basic leucine zipper-like motif
CID: C1-C1 interaction domain
Acidic: Acidic auxiliary domain
NRS: nuclear retention signal
NLS: nuclear localization signal
NES: nuclear export signal

Supplementary Figure S2a

Enhancement of viral growth in A549 cells at 6 hours post-infection, as revealed by increased NP amount in Western Blots.

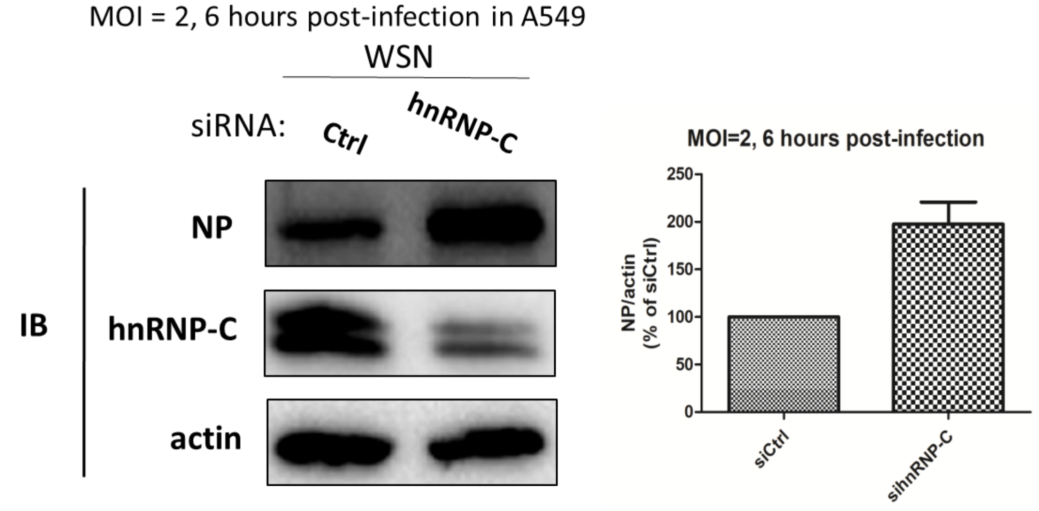

P=0.0018, n=3

Supplementary Figure S2b

Representative Western Blot image for partial depletion of hnRNP-C in HEK293T cells by specific siRNA. Cell lysates were subject to SDS-PAGE and Western Blot after being tested for GFP and luciferase signals, so as to confirm hnRNP-C depletion. HnRNP-C was depleted to 67% on average.

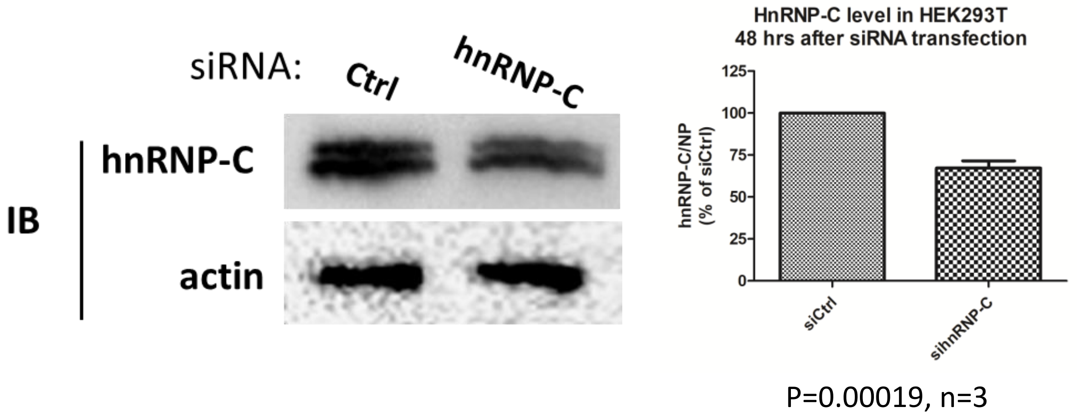

P=0.00019, n=3

Supplementary Table S1
Primers used for cloning hnRNP-C truncations

Clones		Sequences (5' ---- > 3')
A.A. 1-130	Forward primer	CGC ATA CAT ATG GCC AGC AAC GTT ACC
	Reverse primer	ATA CGC GAA TTC TTA TAC ACG TGC TGG GTA ACT G
A.A. 1-217	Forward primer	CGC ATA CAT ATG GCC AGC AAC GTT ACC
	Reverse primer	CGC ATA GCG GCC GC TTA ATT CTT CAT CTC TAC TGC
A.A. 1-251	Forward primer	CGC ATA CAT ATG GCC AGC AAC GTT ACC
	Reverse primer	CGC ATA GCG GCC GC TTA CCC CTC CTC AGC AGA GTC
A.A. 161-293	Forward primer	CGC ATA CAT ATG AAT TCT AAG AGT GGA CAG
	Reverse primer	CGC ATA GCG GCC GC TTA AGA GTC ATC CTC GCC
A.A. 216-293	Forward primer	CGC ATA CAT ATG AAG AAT GAT AAG TCA GAA G
	Reverse primer	CGC ATA GCG GCC GC TTA AGA GTC ATC CTC GCC
A.A. 251-293	Forward primer	CGC ATA CAT ATG GGG GAC CTA CTG GAT GAT G
	Reverse primer	CGC ATA GCG GCC GC TTA AGA GTC ATC CTC GCC

Supplementary Table S2
Microarray analysis revealed upregulation of pro-apoptotic factors and downregulation of anti-apoptotic factors upon knockdown of hnRNP-C in A549 cells.

Gene Symbol	Description	Log 2 C/N	P-value
**Pro-apoptotic factors**			
CASP8	Caspase 8	1.23	5E-05
BIM/ BCL2L11	BCL2-like 11 apoptosis facilitator	1.59	7E-06
BCL2L13	BCL2-like 13 apoptosis facilitator	1.07	3E-06
**Anti-apoptotic factors**			
BNIP3	BCL2/adenovirus E1B 19kDa interacting protein 3	-1.08	5E-07
MDM2	Murine double minute clone 2	-1.01	0.04512
